# Genetic diagnosis of inborn errors of immunity using clinical exome sequencing

**DOI:** 10.3389/fimmu.2023.1178582

**Published:** 2023-05-31

**Authors:** Soon Sung Kwon, Youn Keong Cho, Seungmin Hahn, Jiyoung Oh, Dongju Won, Saeam Shin, Ji-Man Kang, Jong Gyun Ahn, Seung-Tae Lee, Jong Rak Choi

**Affiliations:** ^1^ Department of Laboratory Medicine, Yonsei University College of Medicine, Seoul, Republic of Korea; ^2^ Department of Pediatric Hemato-oncology, Yonsei Cancer Center, Severance Hospital, Yonsei University College of Medicine, Seoul, Republic of Korea; ^3^ Division of Clinical Genetics, Department of Pediatrics, Severance Children’s Hospital, Yonsei University College of Medicine, Seoul, Republic of Korea; ^4^ Department of Pediatrics, Severance Children’s Hospital, Yonsei University College of Medicine, Seoul, Republic of Korea; ^5^ Institute for Immunology and Immunological Diseases, Yonsei University College of Medicine, Seoul, Republic of Korea; ^6^ Dxome, Seoul, Republic of Korea

**Keywords:** inborn errors of immunity, next generation sequencing, clinical exome sequencing, genetic diagnosis, somatic variant, incidental finding

## Abstract

Inborn errors of immunity (IEI) include a variety of heterogeneous genetic disorders in which defects in the immune system lead to an increased susceptibility to infections and other complications. Accurate, prompt diagnosis of IEI is crucial for treatment plan and prognostication. In this study, clinical utility of clinical exome sequencing (CES) for diagnosis of IEI was evaluated. For 37 Korean patients with suspected symptoms, signs, or laboratory abnormalities associated with IEI, CES that covers 4,894 genes including genes related to IEI was performed. Their clinical diagnosis, clinical characteristics, family history of infection, and laboratory results, as well as detected variants, were reviewed. With CES, genetic diagnosis of IEI was made in 15 out of 37 patients (40.5%). Seventeen pathogenic variants were detected from IEI-related genes, *BTK*, *UNC13D, STAT3*, *IL2RG*, *IL10RA*, *NRAS, SH2D1A, GATA2, TET2, PRF1*, and *UBA1*, of which four variants were previously unreported. Among them, somatic causative variants were identified from *GATA2, TET2*, and *UBA1*. In addition, we identified two patients incidentally diagnosed IEI by CES, which was performed to diagnose other diseases of patients with unrecognized IEI. Taken together, these results demonstrate the utility of CES for the diagnosis of IEI, which contributes to accurate diagnosis and proper treatments.

## Introduction

Inborn errors of immunity (IEI) include a variety of heterogeneous genetic disorders in which defects in the immune system lead to an increased susceptibility to infections and other complications. The prevalence of IEI has been known to be rare. However, recent estimated prevalence is 1/1,100 to 1/1,500 and has been increasing over the years with increased recognition of IEI including universal neonatal screening programs in several countries (1, 2).

Defect or dysregulation of the immune system is usually suspected in patients with recurrent or persistent infections, unusual infection, and severe infection. In addition, it is also suspected when recurrent fever and inflammation present. Often, laboratory abnormalities such as low immunoglobulin and abnormal lymphocyte subsets and leukopenia including neutropenia and/or lymphopenia can lead to presumed diagnosis of IEI. Above all, the family history of IEI provides very important clues in suspecting the patient (3).

In the 2022 classification of the International Union of Immunological Societies (IUIS), 485 diseases that affect various components of immune systems are presented. These include immunodeficiencies, antibody deficiencies, complement deficiencies, immune dysregulations, phagocyte defects, and autoinflammatory disorders (4). Most IEI are associated with specific gene defects. When IEI is suspected, it is important to find underlying genetic etiology. Detection of causative pathogenic variants from genes related to IEI is crucial for developing an appropriate treatment strategy and determining the patient’s prognosis.

Because of the wide application of next-generation sequencing (NGS) for detecting genetic variants in IEI, genetic causes of IEI are now more correctly identified and more patients have been diagnosed (5–8). Here, we present the genetic analysis of patients with IEI at a tertiary care hospital in Korea. Genetic causes were identified in 40.5% of the patients (15/37), and four novel pathogenic variants of genes related to IEI were detected. Also, somatic variants were identified in three patients including a patient with VEXAS (vacuoles, E1 enzyme, X-linked, autoinflammatory, somatic) syndrome, which emphasize the utility of genetic test using in-depth NGS, especially clinical exome sequencing (CES) for the diagnosis of IEI.

## Materials and methods

### Subjects

This retrospective study was conducted on patients who received NGS testing for suspected IEI from December 2018 to October 2021 at Severance Hospital, Seoul, South Korea. Their clinical data, clinical manifestations, laboratory test results and genetic analysis results were collected and analyzed.

This study was reviewed and approved by the Institutional Review Board of Yonsei University Health System (4-2022-1558).

### Genetic analysis and variant interpretation

For the genetic diagnosis of IEI, we used a custom-designed clinical exome panel (Dxome, Seoul, Republic of Korea) including 4,894 genes related to human genetic diseases, including IEI (**Supplementary Table S1**). In our panel, we incorporated the majority of the key genes associated with IEI as suggested by IUIS (4); however, 69 genes were not included (see **Supplementary Table S2** for the list of these genes). With our panel, an average depth of 242.6× was achieved (range, 115.7× to 481.5×). Genomic DNA (gDNA) was extracted from peripheral blood of each patient using the QIAamp Blood Mini Kit (Qiagen, Hilden, Germany) according to manufacturer’s guidelines. gDNA was quantified using the Qubit BR dsDNA kit (Invitrogen, Carlsbad, CA, United States). Then, gDNA was fragmented, end-repaired, and ligated to adapters followed by hybridization with probes. Enriched, prepared libraries were sequenced using NextSeq 550Dx instrument (Illumina, San Diego, CA, United States). Alignment, variant calling, and annotation of sequenced data were done by our custom pipeline as described previously (9, 10). Briefly, our study utilized the Burrows-Wheeler Aligner algorithm to map raw sequence data to the GRCh37 (hg19) reference genome. We then processed the data, removing duplicate reads, realigning insertions and deletions, recalibrating base quality, and calling variants using the Genome Analysis Toolkit. Any variants of potential clinical significance were confirmed by visual inspection using Integrated Genomics Viewer (Broad Institute, Cambridge, MA, USA). Large insertions and deletions were detected using split-read approaches with Pindel and Manta algorithms, while structural rearrangements were identified through read-depth analysis using ExomeDepth and combined custom tool (11). Chromosomal copy number variations were cross-checked using a custom pipeline, which included normalizing base-level depth of coverage against other samples in the same batch.

All detected variants were classified based on the recommendation of the American College of Medical Genetics and Genomics (12). For the assessment of pathogenicity, evidence was collected by using population frequency data from multiple databases, 1000 Genomes, the Genome Aggregation Database (gnomAD), the Exome Sequencing Project (ESP), and the Korean Reference Genome Database (KRGDB). *In silico* analysis were conducted by SIFT, MutationTaster, FATHMM, and MetaSVM. Literature and database search for the collection of evidence were conducted using ClinVar, Online Mendelian Inheritance in Man (OMIM), the Human Gene Mutation Database (HGMD), and relevant scientific publications found through online academic databases.

If needed, peripheral blood samples of parents were obtained and tested by Sanger sequencing or CES to determine the *de novo* occurrence of the pathogenic variants (autosomal dominant disorders) or maternal inheritance (X-linked recessive disorders) or whether the suspected variants occurred *in cis* or *in trans* (autosomal recessive disorders), for the confirmation of pathogenicity of detected variants.

### Statistical analysis

For statistical analysis, SPSS version 26 was used (IBM corp., Armonk, NY, USA). Patients with causal variants and without causal variant were compared by Fisher’s exact test, where *p*-values less than 0.05 were considered as significant.

## Results

### Patient characteristics

The demographics of patients included in this study are summarized in **Table 1**. Of the 37 patients who underwent the test, 22 were male (59.5%) and 15 were female (40.5%), aged from 1 month to 64 years old (mean 7.1 and median 2.5 years old). The most common diagnostic category was immune dysregulation (27.0%, 10/37) and periodic fever syndrome (27.0%, 10/37), followed by antibody deficiency (13.5%, 5/37) and combined immunodeficiency (8.1%, 3/37). Twenty-five (67.6%) patients had a history of recurrent or persistent infection. Genetic diagnosis was made in 15 patients (40.5%, 15/37).

**Table 1:** Demographics of patients included in this study (*n* = 37).

	Pathogenic variant detected (*n* = 15)	Pathogenic variant not detected (*n* = 22)	*p*-value
Sex			0.514
Male	10 (66.7)	12 (54.5)	
Female	5 (33.3)	10 (45.5)	
Age (years)			
At symptom onset			0.209
<1	7 (46.7)	7 (31.8)	
1–4	4 (26.7)	9 (40.9)	
5–19	2 (13.3)	6 (27.3)	
>20	2 (13.3)	0 (0.0)	
At diagnosis			0.004
<1	7 (46.7)	4 (18.2)	
1–4	2 (13.3)	11 (50.0)	
5–19	2 (13.3)	7 (31.8)	
>20	4 (26.7)	0 (0.0)	
Category			0.006
Disease of immune dysregulation	6 (40.0)	4 (18.2)	
Periodic fever syndrome	0 (0.0)	10 (45.5)	
Predominant antibody deficiency	3 (20.0)	2 (9.1)	
Combined immunodeficiency	2 (13.3)	1 (4.5)	
Immunodeficiencies affecting cellular and humoral immunity	2 (13.3)	0 (0.0)	
Congenital defects of phagocyte number, function, or both	0 (0.0)	2 (9.1)	
Congenital defects of phagocyte	1 (6.7)	1 (4.5)	
Phenocopies of primary immunodeficiency	1 (6.7)	0 (0.0)	
Defects in intrinsic and innate immunity	0 (0.0)	1 (4.5)	
Auto-inflammatory disorders	0 (0.0)	1 (4.5)	
History of recurrent, persistent infections			0.011
Yes	14 (93.3)	11 (50.0)	
No	1 (6.7)	11 (50.0)	
Family history			0.136
Yes	4 (26.7)	1 (4.5)	
No	11 (73.3)	21 (95.5)	

### Genetic diagnosis of IEI

Detailed clinical characteristics and laboratory test results of patients with genetic diagnosis are shown in **Table 2**. Genetic diagnosis was made promptly after symptom onset in eight cases (60.0%, 9/15). Mean turnaround time of CES for IEI was 33.1 days (range, 13–66 days). In four patients, clinical diagnosis was made several years ago and later confirmed by genetic test (Cases 3, 4, 6, and 14). In two patients, precise diagnosis could not be made before CES, which solved the cases by detecting causative pathogenic variants (Cases 9 and 15). Except for a patient with hypogammaglobulinemia only (Case 1), patients had a history of recurrent or persistent infections. Ten patients had hypogammaglobulinemia.

**Table 2:** Clinical and laboratory data of patients with genetic diagnosis.

Case	Sex	Age		Clinical diagnosis	Clinical characteristics	IgG (mg/dl)	IgA (mg/dl)	IgM (mg/dl)	B cell (%)	T cell (%)	NK cell (%)	CD4+ T cell%^*^ (/µl)	CD8+ T cell%^*^ (/µl)
		Symptom onset	At diagnosis										
1	Male	6M	6M	Hypogammaglobulinemia	–	59 ↓	<5 ↓	11 ↓	0.4	95.8	3.6	69.5 (4,731)	24.4 (1,661)
2	Female	2M	4M	Hemophagocytic lymphohistiocytosis	Recurrent fever and infection (sepsis, meningitis), pancytopenia, splenomegaly	524 ↓	64 ↓	38 ↓	3.4	93.8	2.5	62.7 (3,501)	31.7 (1,770)
3	Female	8Y	33Y	Hyperimmunoglobulin E syndrome	Recurrent fever and infection (liver abscess, cellulitis, cervical abscess, chronic otitis media, breast abscess, pneumonia)	1,491	94	167	NT	NT	NT	NT	NT
4	Female	2Y	7Y	Severe combined immunodeficiency disorder	Recurrent infection, lymph node enlargement, cytomegalovirus infection	477 ↓	<5 ↓	25 ↓	4.3	88	7.1	50.6 (618)	33.8 (413)
5	Male	1M	3M	Severe cytomegalovirus hepatitis	Fever, pancytopenia, sepsis, urinary tract infection, cytomegalovirus infection	<18 ↓	<5 ↓	<5 ↓	89.8	3	5	1.5 (9)	2.1 (13)
6	Male	4Y	37Y	Bruton disease	Recurrent infection (meningitis, skin folliculitis), eczema, bronchiectasis	432↓	<5 ↓	<5 ↓	0	NT	NT	NT	NT
7	Male	1M	6M	Lymphoproliferative disease	Recurrent fever and infection (*Pseudomonas* sepsis and *Clostridium perfringens* acute gastroenteritis), splenomegaly	1,161	91	188	NT	38.6	NT	NT	NT
8	Male	1Y7M	1Y7M	Lymphoproliferative disease	Recurrent fever, bilateral lymph node enlargement, chronic active Epstein–Barr virus infection	927	290.7	256.4↑	NT	NT	NT	NT	NT
9	Female	20Y	22Y	Recurrent nontuberculous mycobacteria pulmonary infection	Recurrent fever and infection (pneumonia, acute pyelonephritis, latent tuberculosis, nontuberculous mycobacteria infection), hepatosplenomegaly	352 ↓	17.3 ↓	10.8 ↓	0	100	0	40.9 (117)	55.4 (158)
10	Female	3Y	3Y	Epstein–Barr virus infection	Chronic Epstein–Barr virus infection	NT	NT	NT	2.3	91.1	6.8	25.5 (584)	60.7 (1,391)
11	Male	1M	1M	Hemophagocytic lymphohistiocytosis	Fever, elevated liver enzymes and bilirubin, hepatosplenomegaly	381↓	6.6↓	14.4↓	6.5	84.6	8.6	0.2 (7)	0.6 (21)
12	Male	5M	5M	*Pneumocystis jirovecii* pneumonia	Pneumonia, recurrent otitis media	423↓	<5↓	26.3↓	99.1	0.7	0.5	NT	NT
13	Male	1M	1M	IL10RA deficiency	Recurrent perianal abscess	343↓	NT	NT	NT	NT	NT	27.4 (238)	43.3 (376)
14	Male	1Y6M	17Y	Bruton disease	Otitis media, sinusitis, *Campylobacter* infection	242↓	<5↓	<5↓	0.1	73.7	NT	43.7 (110)	51.0 (128)
15	Male	61Y	64Y	VEXAS syndrome	Recurrent mycobacterial infection, cytopenia	969	456.2↑	24.9↓	0.4	96.5	3	69.5 (4,731)	24.4 (1,661)

^*^CD4- and CD8-positive T cell % is presented as number of each cell population/total lymphocyte count.

NT, not tested.

In 10 different genes, a total of 17 clinically significant pathogenic variants were found, of which 5 were novel variants (**Table 3**). The most common gene found to have clinically significant pathogenic variants was the *BTK* gene (3/17, 17.6%) followed by the *UNC13D, STAT3*, *IL2RG*, and *IL10RA* genes (2/17, 11.8% for each). The rest included *NRAS, SH2D1A, GATA2, TET2, PRF1*, and *UBA1* (1/17, 5.9% for each). None of the variants were found in common among the patients. Of 17 pathogenic variants, missense variants were most common (6/17, 35.3%), followed by frameshift variants (4/17, 23.5%) and nonsense variants (2/17, 11.8%). A canonical splice site variant, a translation initiation codon variant, a synonymous variant, an intronic variant, and an exon deletion were detected once each.

**Table 3:** Identified pathogenic variants from patients with genetic diagnosis and treatments.

Case	Gene	Accession	Nucleotide	Amino acid	Zygosity (VAF)	ACMG classification	Novel variants?	Disease (MIM#)	Inheritance	Treatment
1	*BTK*	NM_000061.2	c.763C>T	p.Arg255Ter	Hemi	Pathogenic	Known	Agammaglobulinemia, X-linked 1 (300755)	XLR	IGRT
2	*UNC13D*	NM_199242.2	c.1055 + 1G>A	–	Hetero	Likely pathogenic	Known	Hemophagocytic lymphohistiocytosis, familial, 3 (608898)	AR	HSCT
			c.118-308C>T	–	Hetero	Likely pathogenic	Known			
3	*STAT3*	NM_139276.2	c.1144C>T	p.Arg382Trp	Hetero	Pathogenic	Known	Autoimmune disease, multisystem, infantile-onset, 1 (615952) Hyper-IgE recurrent infection syndrome (147060)	AD	Antibiotic prophylaxis
4	*STAT3*	NM_139276.2	c.2144C>T	p.Pro715Leu	Hetero	Likely pathogenic	Known	Autoimmune disease, multisystem, infantile-onset, 1 (615952) Hyper-IgE recurrent infection syndrome (147060)	AD	Ruxolitinib Steroid
5	*IL2RG*	NM_000206.2	c.681del	p.Phe227LeufsTer46	Hemi	Likely pathogenic	Novel	Severe combined immunodeficiency, X-linked (300400)	XLR	HSCT
6	*BTK*	NM_000061.2	c.1780G>A	p.Gly594Arg	Hemi	Pathogenic	Known	Agammaglobulinemia, X-linked 1 (300755)	XLR	IGRT
7	*NRAS*	NM_002524.4	c.38G>A	p.Gly13Asp	Hetero	Likely pathogenic	Known	RAS-associated autoimmune lymphoproliferative syndrome type IV, somatic (614470)	–	Conservative care
8	*SH2D1A*	NM_002351.4	c.1A>G	p.Met1?	Hemi	Pathogenic	Novel	Lymphoproliferative syndrome, X-linked, 1 (308240)	XLR	HSCT
9	*GATA2*	NM_001145661.1	c.1018_1036delinsAATTT	p.Ser340Asnfs*39	Somatic (11.6%)	Likely pathogenic	Novel	Immunodeficiency 21 (614172)	AD	HSCT
10	*TET2*	NM_001127208.2	c.2188del	p.Thr730HisfsTer21	Somatic (9.2%)	Likely pathogenic	Novel	Immunodeficiency 75 (619126)	AR	Ganciclovir Bortezomib
11	*PRF1*	NM_001083116.1	c.65del	p.Pro22ArgfsTer29	Homo	Pathogenic	Known	Hemophagocytic lymphohistiocytosis, familial, 2 (603553)	AR	HSCT
12	*IL2RG*	NM_000206.2	c.340G>A	p.Gly114Ser	Hemi	Likely pathogenic	Novel	Severe combined immunodeficiency, X-linked (300400)	XLR	HSCT
13	*IL10RA*	NM_001558.3	c.537G>A	p.Thr179=	Hetero	Pathogenic	Known	Inflammatory bowel disease 28, early onset, autosomal recessive (613148)	AR	HSCT
		NM_001558.3	Exon 1 deletion		Hetero	Likely pathogenic	Known			
14	*BTK*	NM_000061.2	c.842G>A	p.Trp281Ter	Hemi	Likely pathogenic	Known	Agammaglobulinemia, X-linked 1 (300755)	XLR	IGRT
15	*UBA1*	NM_153280.2	c.121A>C	p.Met41Leu	Somatic^*^ (84%)	Pathogenic	Known	VEXAS syndrome, somatic (301054)	XLR	HSCT

^*^This variant was detected from whole blood sample and was not detected from buccal swab sample.

VAF, variant allele frequency; XLR, X-linked recessive; AR, autosomal recessive; AD, autosomal dominant; IGRT, immunoglobulin replacement therapy; HSCT, hematopoietic stem cell transplantation.

Genetic diagnosis led to appropriate treatment for IEI diagnosed in each patient. Immunoglobulin replacement was done in three patients diagnosed with Bruton disease. Eight patients were treated with allogenic hematopoietic stem cell transplantation.

### Patients with germline pathogenic variants

Pathogenic variants of *BTK* are causes of X-linked agammaglobulinemia, also known as Bruton disease. Known pathogenic variants of *BTK* (Arg255Ter, Gly594Arg, and Trp281Ter) were detected from three patients (Cases 1, 6, and 14) with immunodeficiency. All patients had hypogammaglobulinemia with significantly reduced B-cell number. Of three patients, two patients (Cases 6 and 14) were previously diagnosed with X-linked agammaglobulinemia based on laboratory results and clinical history, while one patient (Case 1) was newly diagnosed by CES performed to identify the cause of hypogammaglobulinemia. The patient did not have clinical symptoms or signs (recurrent infections) suggestive of immunodeficiency.

Two patients were diagnosed with familial hemophagocytic lymphohistiocytosis (fHLH) by CES. In both patients, the laboratory findings, clinical symptoms, and signs suggested HLH. Two pathogenic variants of *UNC13D* were detected from a patient (Case 2) with fHLH. Although HLH was suspected in this patient, HLH was not confirmed because diagnostic criteria (13) were not fulfilled before the molecular diagnosis. In the other patient, Case 11, detection of a homozygous pathogenic variant of *PRF1* confirmed the diagnosis of fHLH, which was made before CES by fulfilling other diagnostic criteria.

A pathogenic *STAT3* variant was detected from an adult female patient (Case 3) with a history of recurrent fever, pneumonia, and breast abscess who was diagnosed with hyperimmunoglobulin E syndrome in childhood. The diagnosis was made based on clinical symptoms and confirmed by the detection of the variant, which was previously reported from patients with hyperimmunoglobulin E syndrome (14, 15). Also, the dominant-negative effect of the variant has been previously observed (14).

A pathogenic *STAT3* variant with several previous reports of autoimmunity and immunodeficiency was detected from a patient (Case 4) with recurrent infection history, lymph node enlargement, pancytopenia, and splenomegaly. A previous study has shown that the variant is a gain-of-function variant (16).

Two novel pathogenic variants of *IL2RG* were identified by CES from patients with severe combined immunodeficiency (SCID). One of the patients (Case 5) had recurrent fever from 2 months old and also had leukopenia, thrombocytopenia, and persistent CMV infection (17). Laboratory findings showed significantly decreased T-cell population and a bone marrow study revealed slightly increased number of histiocytes. Precise diagnosis of the patient was made by genetic test, which revealed a frameshift variant of *IL2RG*. Another novel pathogenic variant of *IL2RG* was detected from a patient with SCID (Case 12). There was a family history of two male brothers of the mother who died of unknown causes when they were young.

A novel, pathogenic *SH2D1A* was detected from a patient (Case 8) with chronic active EBV infection. From 1 year old, the patient had suffered from recurrent fever and bilateral lymph node enlargement. Later, EBV infection was confirmed and persisted despite the treatment.

From an infant (Case 13) with recurrent perianal abscess, genetic diagnosis was made by the detection of two heterozygous pathogenic variants of *IL10RA*. A pathogenic variant (c.537G>A, p.Thr179Thr) was inherited from the mother and *IL10RA* exon 1 deletion was confirmed to occur *de novo*. The silent variant of *IL10RA* has been reported in patients with IL10RA-related inflammatory bowel disease (18, 19). One of these studies has shown the aberrant splicing caused by the variant (18).

In a patient (Case 7) with recurrent fever and infection, genetic testing was requested to find the cause of immune deficiency. A known pathogenic variant of *NRAS* (c.38G>A, p.Gly13Asp) was detected, which caused Ras-associated lymphoproliferative disease.

### Patients with somatic pathogenic variants

Most of the pathogenic variants were germline variants; however, three somatic pathogenic variants were also identified from some patients (3/15, 20.0%).

A somatic *GATA2* pathogenic, frameshift variant (p.Ser340AsnfsTer39, variant allele frequency 11.6%) was detected from a young female adult patient (Case 9) with a history of recurrent infection (tuberculosis, pneumonia, EBV, CMV, and BKV). At initial assessment, the patient had pancytopenia with lymphopenia and monocytopenia. The patient also had B and NK cell deficiency, which was consistent with GATA2-associated immunodeficiency.

A somatic *TET2* pathogenic variant (c.2188del, p.Thr730HisfsTer21, variant allele frequency 9.2%) was detected from a patient (Case 10) with chronic EBV infection. This patient was treated for infectious mononucleosis, and EBV infection persisted despite the treatments. CES, which was requested to evaluate the etiology of chronic EBV infection, detected the somatic variant of *TET2*.

A pathogenic variant of *UBA1*, which is the cause of VEXAS syndrome, was identified by CES from a 64-year-old male patient (Case 15). At initial assessment, the patient suffered from multiple arthralgia, leukopenia, and anemia. During follow-up, chronic granulomatous inflammation with necrosis of bone marrow and tuberculosis lymphadenitis were detected. Various symptoms persisted, however, since the cause was not clearly identified, and CES was performed to diagnose the cause of immunodeficiency and autoinflammatory conditions. A known pathogenic *UBA1* variant (p.Met41Leu, variant allele frequency 84.0%) was detected, which led to an accurate diagnosis of the patient.

### Patients with incidentally identified IEI

There were two patients with incidentally identified pathogenic variants causing IEI, which were identified by CES during genetic workup for other diseases (**Tables 4**, **5**).

**Table 4:** Clinical and laboratory data of patients with incidentally detected inborn errors of immunity.

Case	Sex	Age		Clinical diagnosis	Clinical characteristics	IgG (mg/dl)	IgA (mg/dl)	IgM (mg/dl)	B cell (%)	T cell (%)	NK cell (%)	CD4+ T cell%^*^ (number,/µl)	CD8+ T cell%^*^ (number,/µl)
		Symptom onset	At diagnosis										
Incidental 1	Male	5Y	5Y	Cytopenia	Necrotizing pneumonia, splenomegaly	1,592	284.7	174.5	NT	NT	NT	17.8 (666)	26.2 (981)
Incidental 2	Male	12Y	12Y	Epilepsy	Recurrent lymphadenitis	987	5	17.2	34.9	62	2.9	34.8 (605)	24.4 (424)

^*^CD4- and CD8-positive T cell % is presented as number of each cell population/total lymphocyte count.

NT, not tested.

**Table 5:** Incidental genetic findings and treatment of patients with inborn errors of immunity.

Case	Gene	Accession	Nucleotide	Amino acid	Zygosity (VAF)	ACMG classification	Novel variants?	Disease (MIM#)	Inheritance	Treatment
Incidental 1	*KRAS*	NM_004985.3	c.37G>T	p.Gly13Cys	Somatic (19.6%)	Likely pathogenic	Known	RAS-associated autoimmune leukoproliferative disorder (614470)	AD	Sirolimus Rituximab
Incidental 2	*ISG15*	NM_005101.3	c.379G>T	p.Glu127Ter	Homo	Likely pathogenic	Known	Immunodeficiency 38 (616126)	AR	Conservative care

VAF, variant allele frequency; AD, autosomal dominant; AR, autosomal recessive; HSCT, hematopoietic stem cell transplantation.

CES identified a somatic, pathogenic variant of *KRAS* (c.37G>T, p.Gly13Cys, variant allele frequency 19.6%) from a patient with unknown cause of anemia and thrombocytopenia (Incidental case 1), which made the genetic diagnosis of RAS-associated lymphoproliferative disorder.

To identify a cause of neurodevelopmental disorder, CES was performed for a patient with epilepsy, delayed development, dystonia, spasticity, and brain atrophic changes (Incidental case 2). CES revealed uniparental disomy of chromosome 1 and homozygous *ISG15* pathogenic variants. The patient had a history of CMV infection and recurrent lymphadenitis.

## Discussion

Genetic causes of IEI are increasingly being diagnosed. IUIS have regularly updated the list of IEI, which are caused by single gene defects, and a growing number of genetic defects have been reported (4). Although certain IEI can be suspected through the patient’s history, clinical symptoms, and laboratory tests, it is difficult to make precise diagnosis since there are various genes that cause similar disorders. Thus, NGS, which can test various causative genes at the same time, is very useful for diagnosing IEI.

Diagnostic yield varies among reports, which is due to the difference in the characteristics of study populations and the methods used (**Supplementary Table S3**). Previous studies used targeted panel sequencing (20, 21), CES (22), whole exome sequencing (WES) (23, 24), or whole genome sequencing (25), and each method has its pros and cons. With targeted gene panel sequencing, in-depth sequencing of selected genes related to IEI is possible, which enables detection of low-level mosaic variants, whereas with methods that can test a large number of genes, diagnostic yield would be increased. In addition, as well as newly identified genes related to IEI, previous unknown related genes can be assessed. In this study, CES was used for genetic diagnosis, which showed a 40.5% positive rate. Pathogenic variants of a gene not included in targeted panels used in other studies (20, 21), *UBA1*, were detected by CES. In addition, two mosaic variants with low variant allele frequency were successfully detected. Taken together, these showed the utility of CES for diagnosis of IEI.

For patients with IEI, early diagnosis is crucial for proper management and avoiding severe, recurrent infections. In our cases, genetic diagnosis was made in a few months after symptom onset in patients younger than 2 years old, which emphasizes the clinical utility of CES for the diagnosis of IEI. CES is also useful for the diagnosis of unsolved cases. Two patients with a long history of undiagnosed recurrent infection and inflammations were diagnosed by CES after 2 years and 3 years after the onset of symptoms, respectively, which led to appropriate curative therapy for the patients. In particular, in the case of patients with *GATA2* and *IL10RA* variants, prior to genetic diagnosis, the current ongoing infection treatment was given priority, but after genetic diagnosis, allogeneic hematopoietic stem cell transplantation, a curative treatment option, could be attempted with more confidence.

Although disorders of the immune system can be suspected in patients with various conditions, diagnostic yield seemed to be different among conditions. Based on the clinical diagnoses and clinical features of patients without a genetic diagnosis, it appears that they tend to have a milder disease or exhibit a lower frequency of infections (**Supplementary Table S4**). Recurrent fever can be evidence of immune disorder; however, in our study population, genetic analysis did not yield any positive result from the patients with recurrent fever without apparent infections. Several diseases were associated with periodic fever syndrome, such as cryopyrin-associated periodic syndromes (caused by *NLRP3*), familial Mediterranean fever (caused by *MEFV*), tumor necrosis factor receptor-associated periodic syndrome (caused by *TNFRSF1A*), and mevalonate kinase deficiency (caused by *MVK*) (26). Our patients were diagnosed with PFAPA syndrome, by combining clinical features and negative genetic test results. Although there might be unknown genetic causes, genetic testing for patients with periodic fever without identifiable infection is more likely to yield negative results.

Previous studies have reported the presence of mosaic variants in patients with various IEI (27, 28). For example, a somatic, heterozygous pathogenic variant of *FAS* is a known cause of autoimmune lymphoproliferative syndrome (29). There was a recent report of mosaic *TLR8* gain-of-function variants that cause immunodeficiency (30). In this study, we also revealed three patients with somatic pathogenic variants of *GATA2, TET2*, and *UBA1*. Pathogenic variants of *GATA2* were known to cause immunodeficiency with variable onset and susceptibility to mycobacteria and fungal infections (31). The patient with the *GATA2* variant in this study also showed similar features, with a history of recurrent infection with mycobacterial infection, hypogammaglobulinemia, and B and NK cell deficiency. Somatic pathogenic variants of *TET2* have been reported from patients with various hematologic malignancies (32). Although the association between *TET2* variant and IEI has not been elucidated yet, a recent study reported that somatic *TET2* variants were detected in CD4+ T lymphocytes of a patient with combined variable immunodeficiency (33). The *UBA1* somatic variant was detected from a patient with a long history of recurrent infections and systemic inflammation of unknown etiology, whose diagnosis of VEXAS syndrome was made by CES. VEXAS syndrome is an adult-onset autoinflammatory disease defined recently, caused by myeloid lineage restricted somatic variants of *UBA1* (34). Since various studies have revealed the presence of low-level mosaic variants and variants detected only in specific population of peripheral mononuclear cells, simple, routine genetic analysis without cell sorting or in-depth sequencing can lead to missed detection of some variants related to IEI.

Among the known indicators that suggest the presence of IEI, a recurrent and/or persistent history of infection and a family history of IEI have been previously reported (35–37). The results of this study are consistent with these earlier findings, demonstrating a correlation between infection history and IEI (35, 36). However, the relationship between family history and IEI remains a subject of debate, with some studies reporting a connection and others finding no association (35–37). In this research, no statistically significant correlation was found between family history and molecular diagnosis of IEI. Nevertheless, a higher proportion of cases with a family history of IEI were observed among those who had a genetic diagnosis. This observation suggests that family history may be a useful factor to consider when deciding whether to perform genetic testing for IEI and for supporting the diagnostic process, potentially providing valuable information for a more comprehensive understanding of the patient’s condition.

In this study, we also described patients with IEI, who were diagnosed incidentally. Owing to CES, which was used for genetic diagnosis for various diseases in our institute, IEI could be diagnosed in previously unrecognized patients. This shows the benefits of using CES for various disease groups as well as IEI.

In summary, this study showed the clinical utility of CES for the diagnosis of IEI. In addition to pediatric patients suspected of primary immunodeficiency, early CES should be considered in adult patients with suspected symptoms that may be caused by immunodeficiency or immune dysregulation. Early, accurate diagnosis made by CES results in the reduction of labor and cost for other diagnostic methods and can improve patient’s prognosis and quality of life by reducing time before diagnosis is made.

## Data availability statement

The datasets presented in this study can be found in online repositories. The names of the repository/repositories and accession number(s) can be found below: SCV003837562 - SCV003837580 (ClinVar).

## Ethics statement

The studies involving human participants were reviewed and approved by Institutional Review Board of Yonsei University Health System. Written informed consent from the participants’ legal guardian/next of kin was not required to participate in this study in accordance with the national legislation and the institutional requirements.

## Author contributions

Conceptualization: SK, SS, and J-MK. Data curation: SK, YC, JO, DW, SS, and J-MK. Funding acquisition: SS and J-MK. Investigation: SK, YC, SH, JO, SS, J-MK, JA, S-TL, and JC. Methodology: SK, JO, DW, SS, and J-MK. Project administration: SS, J-MK, JA, S-TL, and JC. Supervision: SS and J-MK. Writing—original draft: SK. Writing—review and editing: SK, YC, SH, JO, DW, SS, J-MK, JA, S-TL, and JC. All authors contributed to the final article and approved the submitted version.
