# Corrigendum: Genetic diagnosis of inborn errors of immunity using clinical exome sequencing

**DOI:** 10.3389/fimmu.2023.1235318

**Published:** 2023-06-19

**Authors:** Soon Sung Kwon, Youn Keong Cho, Seungmin Hahn, Jiyoung Oh, Dongju Won, Saeam Shin, Ji-Man Kang, Jong Gyun Ahn, Seung-Tae Lee, Jong Rak Choi

**Affiliations:** ^1^ Department of Laboratory Medicine, Yonsei University College of Medicine, Seoul, Republic of Korea; ^2^ Department of Pediatric Hemato-oncology, Yonsei Cancer Center, Severance Hospital, Yonsei University College of Medicine, Seoul, Republic of Korea; ^3^ Division of Clinical Genetics, Department of Pediatrics, Severance Children’s Hospital, Yonsei University College of Medicine, Seoul, Republic of Korea; ^4^ Department of Pediatrics, Severance Children’s Hospital, Yonsei University College of Medicine, Seoul, Republic of Korea; ^5^ Institute for Immunology and Immunological Diseases, Yonsei University College of Medicine, Seoul, Republic of Korea; ^6^ Dxome, Seoul, Republic of Korea

**Keywords:** inborn errors of immunity, next generation sequencing, clinical exome sequencing, genetic diagnosis, somatic variant, incidental ﬁnding

In the published article, there was an error regarding the affiliation for Seung-Tae Lee and Jong Rak Choi. As well as having affiliation 1, they should also have Dxome, Seoul, Republic of Korea.

In the published article, there was an error. In **Materials and Methods**, the manufacturer of our custom panel was missed.

A correction has been made to **Materials and Methods**, “*Genetic analysis and variant interpretation”*, paragraph 1. This sentence previously stated:

“For the genetic diagnosis of IEI, we used a custom-designed clinical exome panel including 4,894 genes related to human genetic diseases, including IEI (Supplementary Table S1).”

The corrected sentence appears below:

“For the genetic diagnosis of IEI, we used a custom-designed clinical exome panel (Dxome, Seoul, Republic of Korea) including 4,894 genes related to human genetic diseases, including IEI (Supplementary Table S1).”

